# Moralisation, stigmatisation, and downstream interventions: How symbolic violence sustains geographic inequalities in deaths of despair in England

**DOI:** 10.1016/j.socscimed.2025.118451

**Published:** 2025-07-25

**Authors:** Timothy Price

**Affiliations:** Population Health Sciences Institute, Faculty of Medical Sciences, Newcastle University, Newcastle upon Tyne, NE1 4LP, UK

## Abstract

This paper examines how symbolic violence contributes to the persistence of geographic inequalities in deaths of despair (DoD), those from drug, suicide, and alcohol-specific mortality, in two deindustrialised towns in North East England. Although there is evidence that DoD are driven by structural determinants, such as economic policies, inadequate social safety nets, and poverty, less is known about how these determinants are ideologically obscured. Drawing on qualitative data collected from 30 residents of Middlesbrough and South Tyneside, two towns with above average rates of DoD, the analysis explores how symbolic violence, in the form of moralisation, stigma, and downstream interventions, frames behaviours as individual failings, downplays structural determinants, and sustains inaction while maintaining the appearance of intervention. By diverting attention away from the root causes of DoD, symbolic violence functions as a mechanism through which these inequalities are reproduced. By highlighting the cultural and ideological processes that allow structural determinants to go unaddressed, this study deepens our understanding of how inequalities in these deaths are sustained and legitimised in marginalised places. The findings point to the need for future interventions to not only improve material conditions but also critically engage with the symbolic forces that reinforce geographic disparities in DoD.

## 1 Introduction

Over much of the past century, high-income countries have experienced remarkable gains in life expectancy, largely due to improvements in healthcare, living standards, and public health interventions (Ho and Hendi, 2018). Yet, in the mid-2010s, this long-standing trend began to falter. Although most high-income nations quickly resumed the trend of increasing life expectancy, the United States (US) and the United Kingdom (UK) emerged as notable exceptions. Economists Anne Case and Deaton (2015, 2017, 2020) identified a key factor behind declining US life expectancy: increasing rates of death among non-Hispanic white Americans aged 45–54 from drug, suicide, and alcohol specific mortality. Case and Deaton (2020) termed deaths from these causes ‘deaths of despair’ (DoD) and proposed that long-term shifts in social structures and labour market conditions, such as delayed marriage, increased childcare demands, limited educational opportunities, stagnant wages, and rising housing costs had created cumulative disadvantage over successive generations, fostering widespread despair that ultimately contributed to increasing rates of DoD. Case and Deaton’s (2020) proposition that DoD share a common underlying cause has been the subject of ongoing debate (Ruhm, 2021; Darke et al., 2025; Plunk et al., 2018; Lindstrom, 2023). Nonetheless, their work has significantly influenced both academic discussions and media coverage. The term ‘deaths of despair’ is now widely used in scholarly articles and news reports (Aspholm, 2022; Beseran et al., 2022; Walsh et al., 2021; Dowd et al., 2023; Van Dam, 2023; Peterson and Densley, 2023).

Initially thought to be a uniquely American issue, rising rates of DoD have also attracted research attention in the UK (Leon et al., 2019; Dowd et al., 2023; Camacho et al., 2024; Koltai et al., 2020; Walsh et al., 2021). Although DoD only partially explain the stalling life expectancy in the UK prior to the COVID-19 pandemic (Ho and Hendi, 2018), there has been a notable rise in DoD rates in the UK since the early 2000s (Augarde et al., 2022). Much of this increase has been driven by increases in drug-related mortality, with deaths from suicides and alcohol-specific causes increasing at a more moderate rate (Dowd et al., 2023; Augarde et al., 2022). The existing literature from both the US and UK surrounding the factors underlying increasing rates of DoD has found an association with structural determinants such as welfare, housing, and labour market policies (Dow et al., 2020; Bradford and Bradford, 2020; Bjorklund, 2023). There are also clear associations between increased rates of DoD, poverty and economic decline with deaths from these causes concentrating in deindustrialised, low-income communities in England (Camacho et al., 2024; Augarde et al., 2022) and other national contexts, such as the US (Herzog, 2020; Chetty et al., 2016) and Eastern Europe (King et al., 2022; Scheiring and King, 2023).

There are significant geographic inequalities in rates of DoD within the UK; for example, Scotland has a significantly higher rates of DoD than other constituent countries (Walsh et al., 2021; Allik et al., 2020). Within England, geographic inequalities in rates of DoD persist between regions and cities and towns within regions (Camacho et al., 2024; Augarde et al., 2022). In the North East, the English region with the highest rates of DoD, two local authorities, Middlesbrough and South Tyneside have significantly higher than regional and national average rates of DoD (ONS, 2023a, 2023b, 2024). These geographic patterns reflect broader social and economic inequalities that are deeply embedded and are produced and sustained through a combination of structural and social determinants (Price and Mcgowan, 2025). Understanding why these inequalities persist requires not only examining material conditions but also considering how cultural forces shape perceptions of health inequality. One concept that offers valuable insight into this process is Pierre Bourdieu’s theory of symbolic violence (Bourdieu, 1977).

Sociologist Pierre Bourdieu introduced the concept of symbolic violence to describe how systems of power and domination are maintained through cultural means rather than direct physical force (Bourdieu, 1977). According to Bourdieu, dominant groups shape the norms, values, and beliefs of subordinate groups via language, education, and institutional structures, leading those in less powerful positions to view existing social conditions as just and legitimate (Bourdieu, 1977, 1990). Symbolic violence highlights the subtle, often invisible ways that power permeates everyday life, influencing how people think, act, and accept existing social orders without the need for obvious coercion. Importantly, symbolic violence does not require conscious intent; it is often reproduced inadvertently through taken-for-granted norms, language, and institutional practices. In public health research, symbolic violence has been employed to critique health promotion efforts that individualise blame for poor health outcomes, thereby ignoring the structural determinants that contribute to ill health (Lupton, 2015; Mikkelsen et al., 2021). While not always framed explicitly as symbolic violence, several studies have also illustrated how marginalised groups internalise cultural narratives surrounding illness, blame themselves for their poor health, and come to see systemic deprivation as personal failure (Thomas et al., 2020; Llewellyn-Beardsley et al., 2023; Peacock et al., 2014). This existing research highlights how symbolic violence works through processes of obfuscation, where structural causes are rendered invisible, and internalisation, where those affected by inequality accept dominant explanations that legitimise their suffering.

In this study, I build on this body of work by showing how symbolic violence operates in the context of DoD in post-industrial communities in England. As in previous studies, I find that the political and economic determinants of ill-health are obscured through narratives that emphasise individual responsibility and moral failure. This obfuscation helps to naturalise geographic inequalities in health, making them appear inevitable or deserved, and thereby sustaining the conditions that produce them.

## 2 Methods

The findings reported in this article are derived from a broader research project examining stakeholder and community member explanations for geographic inequalities in rates of DoD; findings unrelated to symbolic violence in community member narratives are reported elsewhere (Price, 2024, 2025; Price et al., 2024). This paper focuses on community member narratives to explore how symbolic violence sustains geographic inequalities in DoD. While stakeholders offer institutional perspectives, community members provide unique insight into how cultural framings of responsibility, stigma, and neglect are lived and reproduced within affected places.

### 2.1 Recruitment and data collection

30 community members participated in this study. The participants were recruited from Middlesbrough and South Tyneside. Participants were recruited from community groups that were open to the general public in their respective towns. These groups were intended to provide community and social connection, rather than peer support for a specific challenge. The study was also advertised by flyers displayed in community hubs (e.g. libraries, community centres) and in organisations frequented by some members of the community (e.g. charity services). People were eligible to participate in this study if they were over the age of 18, were a resident of Middlesbrough or South Tyneside, were comfortable discussing DoD, and were able to complete an interview in English. Table 1 provides demographic information for participants in this study.

Participants in one recruitment location requested to do their interviews as a group, to accommodate that request, data were collected from these participants via a focus group (n = 6). All other data were collected via semi-structured interviews (n =24). The interviews and focus group were conducted using a bespoke topic guide that explored how a range of factors, including mental health, poverty, and government policy may contribute to local rates of DoD. Data collection ended when the author deemed that data saturation had been reached.

Participants provided informed consent to participate and for their anonymised data to be used in research outputs prior to participating in the study. Participants were provided with a £25 supermarket voucher to thank them for their participation. Participants shared a range of critical views of local services and institutions, as well as deeply personal stories and experiences. As such, participant data is confidential, and anonymised transcripts are not publicly available. Direct quotations used in this publication are not attributed to specific participants to further mitigate the risk of identification.

### 2.2 Analysis

All interviews and the focus group were audio-recorded and transcribed by the researcher. A coding matrix was deductively generated based on the topic guide used during data collection and supplemented with inductively generated codes as coding progressed. The coding matrix and a sub-sample of coded transcripts were reviewed by another researcher within the author’s research institute to ensure reliability. Data analysis was guided by the Iterative Categorization (IC) technique developed by Neale (2016) and findings were interpreted through thematic analysis (Braun and Clarke, 2006). IC is a technique for managing data analysis, rather than an analytic method in and of itself (Neale, 2016). IC is rigorous, transparent, and compatible with common forms of qualitative analysis, such as thematic analysis (Neale, 2016); it has been used to support analysis in previous studies on addiction (Neale et al., 2012, 2017). One stage of IC requires the researcher to “differentiate” their data. The differentiation process involves checking themes for differences and similarities based on participant subgroups to investigate whether participants who expressed similar beliefs share discernible characteristics. In this study, participant data was differentiated based on age group, gender, and area of residence. After the differentiation process was completed, few differences in participant narratives based on gender or town of residence were identified. The similarity between participant narratives regardless of demographic groups indicates a high degree of consensus among participants and justifies viewing their data as that of a single group.

## 3 Findings

Three themes provide insight into the role that symbolic violence plays in obfuscating the determinants of DoD and sustaining the geographic inequalities in rates of these deaths present in participants towns: Moral Judgements and Explanations, Stigmatisation and Neglect, and Inadequate Services and Downstream Interventions.

### 3.1 Moral judgements and explanations

A common theme in participants’ narratives was the belief that people who experience drug, alcohol, or suicide-related harm are responsible for their own circumstances. While many participants acknowledged economic challenges, some still felt that people ultimately had a choice in whether or not to engage in harmful behaviours. This theme is particularly evident in a quote from a participant in Middlesbrough.

“You’ll tell me its cause of poverty, That’s all a load of rubbish that. I mean, if you don’t want to take drugs you don’t have to. I only had one cigarette when I was 14 and I never had another one since. It’s up to you if you don’t want to take drugs. You don’t have to take them. You don’t have to go drinking if you don’t want to. Don’t have to do anything. It’s a load of rubbish trying to blame that.” - Middlesbrough

This quote shows a clear rejection of structural explanations for above average rates of DoD in Middlesbrough. For this participant, drug use was a personal decision, unrelated to social or economic conditions. As they saw it, since people decided to initiate drug use, they were responsible for the problems that arose as a result; thus, addiction and drug related deaths were solely the products of poor choices. Some participants also extended opinions about individual responsibility to mental health, blaming people who lived with depression and anxiety for not trying hard enough to overcome their poor mental or for engaging in behaviours that make their mental health worse.

“It seems to me that virtually everyone has anxiety, has depression. We’re not talking ADHD, autism, we’re talking about depression and anxiety. I’m like, everybody? Really? Again, that seems to be becoming the norm as well. It wasn’t shouted about as much in my day. But everybody’s got anxiety, it’s just how you deal with it. I think as well that a lot of people say “I’ve got this, I’ve got that.” It just allows them not to work, not take care of themselves. If it’s so bad and stopping you from getting employment … do something to start feeling better about yourself, stop drinking and smoking [*cannabis*] and you’ll bring money in and have a routine and be feeling better. But they’re very good at saying “cant do because of anxiety” and I think its being overused.” – South Tyneside

A participant in Middlesbrough extended this line of thinking to people who have died by suicide. When asked why Middlesbrough had an above average rate of suicides, the participant explained that most deaths by suicide were related to the use of cocaine.

“People have got terrible cocaine addictions. That’s one of the things I think is causing a lot of suicide here. It seems to be, there’s a massive link. Every other week a young man is killing themselves. It all links back to cocaine addiction. My friend from school, her partner hung himself in the local park that his children go in because he had been on a cocaine bender. Lovely lad, just a cocaine addiction.” – Middlesbrough

This kind of thinking reflects the way that narratives of individual responsibility surrounding the causes of DoD have become embedded in society, including in places like Middlesbrough and South Tyneside where rates of DoD are among the highest in the country. In each of the examples above, participants described drug use, mental health problems, and suicide as a product of personal failings or individual choices. As a result, the focus remains on the individuals who are at risk of, or have died by, DoD, rather than the structural determinants of such deaths.

### 3.2 Stigmatisation and neglect

Participants expressed the belief that their towns were stigmatised. They felt that places like Middlesbrough and South Tyneside were viewed through a negative lens and were associated with poverty, drug addiction, and criminal activity. This reputation, participants noted, was entrenched in the public imagination and was reinforced by media portrayals and public discourse which focused on negative aspects of the town. Many participants felt that these perceptions were intensified by the visible presence of issues such as homelessness and open drug use in public spaces which were described as particularly visible to visitors and therefore thought to play a significant role in shaping external opinions.

“When I moved here people are like “oh, why are you moving to South Tyneside? It’s full of druggies.” It doesn’t, it’s not a great representation. It just sends the message it’s just full of druggies, and it’s not. It’s just one problem, every town’s got them. But you come out the metro station and there is always people with issues hanging around. It’s been mentioned to me a few times to me that it’s the first thing you see.” – South Tyneside

Participants in this study reported that representations of their areas in political discourse and news media had further entrenched the sense of stigmatisation in their towns. This was particularly common in Middlesbrough, with participants citing the Channel 4 show *Benefits Street*, which was filmed in the area, and (then Secretary of State for the Home Department) James Cleverly’s comments in which he described Stockton-on-Tees (a deprived town that borders Middlesbrough) as a “shit-hole” in late 2023.

“When you mention you’re from Middlesbrough you get “oh smoggies.” That’s a nickname for people from Middlesbrough back when it was very industrial with all the smoke. It’s seen as a really deprived place. It’s been on some quite nasty TV shows where people were portrayed really negatively. It’s called Benefits Street, and it was in Middlesbrough. Stockton as well. So, we get a lot of negative portrayal. A few years ago one of the newspapers had Middlesbrough as the worst place in the UK to live.” – Middlesbrough

Participants told me that people from low-income communities like Middlesbrough, South Tyneside, and others were lumped together, and people applied their judgements about the town to the individuals who lived there. Participants in this study indicated that growing up in a stigmatised community instilled a sense of shame in residents that affected them for the rest of their lives. According to participants, people internalised the stereotypes about their community and people living in poverty and believed them to be true about themselves. Participants believed that this negatively affected one’s outlook and general wellbeing.

“If someone is always telling you the place you live is rubbish, it’s going to affect you. You’re going to think “it’s a rubbish place to live, I must live here because I’m rubbish too”. – South Tyneside

Participants believed that feelings of alienation from broader society created resentment for people in other parts of the country. The sense of shame described by some participants was visible in other participants’ accounts of their experience living in the community. Participants expressed embarrassment, shame, and anger about the fact that they live in a town that is so looked down upon.

“I’m sorry. I am, I’m sorry I’m from Middlesbrough. I’m ashamed to say I’m from Middlesbrough. That’s why I went to [*a different country*]. If it wasn’t for [*personal circumstances*] I would never have come back here.” – Middlesbrough

Participants explained that the stigma surrounding their towns changed how residents viewed themselves and their lives. They believed that experiences that were routine or unexceptional for many in English society, such as pursuing further education after school, career advancement, and obtaining a source of stable income were seen as out of reach for many people in their towns. This divide between the “haves” and the “have nots” was described by some participants and could be observed in other participants’ accounts of their own lives. When a participant from South Tyneside was describing the job market in her town, she consistently described a two-tiered society in which there were jobs available for “them”, but not for “us”. This two-tiered nature of society was so fundamental to her experience and worldview that she struggled to articulate it to me, but it was clearly based along class lines.

“**Participant**: … so, there is a lot going on in South Tyneside, but people like us, we get affected by it most because we can’t get a job in them sectors. Even if we’ve got experience.

**Interviewe**r: You’ve said that a few times now, “people like us”. What do you mean by that? **Participant**: Like me. Like, I’m in a major financial crisis. I’m living on the dole.

**Interviewer**: But who is “us”? Or maybe, who is “them”?

**Participant**: I… I don’t know, really, like how else to say it.”

-South Tyneside

Participants reported that the stigmatisation of their communities resulted in people in other parts of the country, and members of the Government, not caring about the problems in their towns, such as the lack of economic opportunity and level of poverty, that they believed contributed to the above average rates of DoD. They described the Government and people in the south of the country more generally as out of touch with the needs of their towns. It should be noted that participants did not specify that these feelings were directed at the then current Conservative Government, rather that they were speaking about government more broadly. According to these participants, the out of touch nature of the Government resulted in national policies that benefited the South while failing to address the needs of Northern communities. In this way, participants saw the stigmatisation of their communities as a force sustaining government neglect of deprived communities.

“I just feel because the Government is in London, they think, I think, the Government thinks the North East is all just Manchester somewhere. They don’t seem to understand. Even when they come up here, it’s just for a photo opportunity. No one really knows what everyday life is like. They don’t come to community projects or actually see the people. It’s just such a waste.” – Middlesbrough

### 3.3 Inadequate services and downstream interventions

Participants described a range of services that were available to support people in poverty or those suffering from addiction or mental health problems, but reported that such services were rarely effective, and were sometimes harmful. Participants explained that mental health services often failed to adequately support people in need due to a range of limitations inherent to their design. For example, it was reported that many mental health services would not provide support to people experiencing co-occurring substance use and mental health disorders if the person was still using substances. Participants felt that addiction often coincided with poor mental health, which left people in a cycle where they could not combat their addiction because of their mental health but could not get support for their mental health because of their addiction. Participants felt that this design, which they viewed as inherent to the treatment landscape, meant that mental health and substance use treatment services set people up to fail when seeking recovery.

“It’s counterproductive because I know to get counselling you have to be free of drugs and drink, but at the same time, that could be the issue that’s causing the drugs and drink. It feels like, to me, surely, it’s not ideal to go to sessions drunk, but if you could solve that problem maybe they wouldn’t be drinking anyway. Surely instead of just trying to put a bandage over it you should be going to try and fix it.” – Middlesbrough

Other limitations to mental health service effectiveness included long wait times and difficulty securing appointments. Several participants reported that their mental health worsened while they waited for treatment from healthcare providers.

“Even your GP. Your GP is hard enough to find now. If you call your GP, they just want to speak to you on the phone. Good luck if you want to actually meet them, you’ll wait years. I had about two mental breakdowns before I had an appointment.” – Middlesbrough

The benefits system was believed to offer inadequate support for people living in poverty. Participants felt that benefits were an important social support, but that the system was often difficult to engage with and that the benefits one could receive were often insufficient to help them escape poverty. It was reported by participants that applying for benefits was complicated and confusing. When people were able to access benefits, participants reported that the system could be degrading and stigmatising and often applied sanctions arbitrarily; it was believed that these effects negatively impacted the mental health of people claiming benefits.

“I hear of people being sanctioned for absolutely bizarre reasons. For having an appointment in hospital that they couldn’t change for example. It was either the hospital appointment or the benefits appointment, they chose hospital and got sanctioned. Where is the justice in that? The benefits system is … Sanctions are meant to frustrate people, so they start looking to go back to work, but it’s having the opposite effect. It’s locking people into poverty. They spend all their time just avoiding sanctions so they can’t think of ever doing anything else. They have to do all sorts of ridiculous things which is terrible for their self-esteem. How does that make any sense at all? Then you’re sat at home with a load of problems, hating yourself and thinking you’re of no use to anyone.” – Middlesbrough

In the absence of adequate income support, participants believed that people in poverty turned to charity services, such as foodbanks and community kitchens to provide for their basic needs. While there was agreement that these charity services met important needs in the towns, some participants had concerns about the prevalence of foodbank reliance in their areas. These participants believed that food banks were emblematic of the government’s failure to alleviate poverty and support people on low incomes.

“In my opinion, we shouldn’t have a foodbank, we shouldn’t ever have a foodbank. But we rely on them a lot. We, you know, in this country, nobody should be homeless. Nobody should be without food. But we’re in the position where we have to have those things. We’ve got the wealth in this country to provide for everyone very comfortable [*sic*], but we choose not to, or some people choose not to. We’re just papering over.” – South Tyneside

Even larger policy efforts, such as the Levelling Up agenda, aimed at reducing regional economic disparities by investing in infrastructure, education, and local services to boost growth and opportunities in underperforming areas outside London and the South East, were seen by participants to be inadequate and a distraction from historic neglect of the North.

“I think that’s part of the levelling up fund. I see that £3 million as a token gesture, “there you go, keep your mouth shut because actually, over the last ten years you haven’t had a penny.” How can you change something that you know is going to take ten years before you start to see any fundamental real change when your investment process allows you one year of investment? If we’re looking to change something ‘round here, it’s going to take a very long time.” – Middlesbrough

## 4 Discussion

The findings of this study show how symbolic violence helps to sustain geographic inequalities in deaths of despair by shaping how suffering is understood, judged, and responded to. Across the three themes. moral narratives, territorial stigma, and inadequate services, symbolic violence functions as a cultural mechanism that obscures structural drivers of inequality and reinforces the status quo. First, moralised understandings of addiction, mental illness, and suicide align with dominant cultural logics that attribute despair to personal weakness rather than structural harm. Second, territorial stigma operates as symbolic violence made spatial, positioning entire communities as inherently broken or undeserving, and legitimising ongoing disinvestment. Finally, minimal and punitive welfare services, along with charitable interventions like foodbanks, create the appearance of care while offering little meaningful change, redirecting attention away from structural change towards limited intervention. Taken together, these forms of symbolic violence work to naturalise inequality, individualise blame, and narrow the possibilities for public and political responses to despair.

### 4.1 Individualisation and moralisation of DoD

In the context of health and disease, moralisation refers to the process through which neutral actions (those that are held to be neither morally good nor bad) are assigned moral significance (Kraaijeveld and Jamrozik, 2022; Rozin et al., 1997). Of particular relevance to the findings of this study is the moralisation of substance abuse and mental illness, which empirical research has shown to be heavily moralised conditions (Frank and Nagel, 2017; Haslam et al., 2007; Salter and Breckenridge, 2014; Brown and Tucker-Seeley, 2018). The moralised views of drug, suicide, and alcohol-specific morbidity and mortality that were present in participant narratives in this study reveals an important function of symbolic violence in the context of DoD. In participant narratives, addiction, suicide, and poor mental health were often interpreted not as responses to deprivation, but as evidence of weak character or moral failure. These narratives reflect more than isolated personal beliefs; they mirror dominant cultural logics that have become deeply embedded in public discourse and social institutions. In this sense, symbolic violence is evident not because participants are inherently “wrong” to attribute DoD to individual actions (indeed, there is an unavoidable element of individual choice in substance abuse and self-harm), but because their explanations for inequalities in these deaths align with, and reproduce, ideological frameworks that obscure structural causes and attribute blame entirely to the individual. Whether these beliefs are rooted in participants’ lived experience in communities affected by DoD or internalised from broader social narratives surrounding these causes of death is ultimately immaterial, their effect is the same: they perpetuate symbolic violence by individualising despair and deflecting attention from its structural determinants.

These narratives position DoD as products of individual choices, rather than as social consequences, effectively disqualifying them from being treated as a collective or systemic issue (Goodyear, 2021; Ben-Yehuda, 1990). Through these narratives, symbolic violence positions DoD not as outcomes of systemic neglect, but as the predictable consequence of flawed individuals, thereby legitimising the inequitable conditions that produce them. This framing reflects a long-standing moral tradition in English social thought, one that stretches back to the 19th century and the ideological foundations of the Elizabethan Poor Laws (Barry and French, 2004). Much like the Victorian distinction between the “deserving” and “undeserving” poor, the narratives in this study surrounding addiction, suicide, and mental illness cast these issues as the consequences of individual weakness, lack of discipline, or moral failure. These Dickensian ideas continue to shape how suffering is understood and responded to and are a manifestation of symbolic violence that frames DoD as issues of morality. This is a reflection of how political and institutional narratives become taken-for-granted truths. In this way, symbolic violence is enacted when individuals adopt and reproduce these narratives as common sense, despite their own proximity to the harms being misrepresented. This dynamic aligns with Bourdieu’s (1977) concept of doxa, the deeply internalised norms and assumptions that make power relations appear natural, inevitable, and beyond question (Deer, 2008; Eagleton and Bourdieu, 1992). Doxa does more than shape what people believe; it shapes what feels sayable, reasonable, or morally coherent. In this context, Dickensian ideas of moral failure, self-discipline, and personal responsibility are not just available narratives, they are the ones culturally reinforced through policy, media, and everyday discourse. Structural explanations, while materially valid, may be harder to access or sustain because they fall outside what doxa presents as obvious or legitimate.

The moralisation of DoD can be seen to serve two purposes. First, it delegitimises the suffering of those affected by these mortalities and related morbidities and deflects responsibility away from the state and society. By attributing blame to individuals, symbolic violence allows the broader political and economic systems that foster inequality to remain unchallenged. This view is consistent with existing critiques in the literature surrounding the intersection of neoliberalism and health (Schrecker and Bambra, 2015; Cooper and Whyte, 2017), which argue that framing poverty and poor health as personal failures is a key ideological tool that justifies austerity and minimal social intervention. Within this context, DoD are not seen as issues of injustice that necessitate systemic reform, but as unfortunate yet avoidable outcomes, effectively rendering them non-issues in public discourse. There is precedence in the existing DoD research to support the view that public discourse surrounding DoD focuses on individual behaviours while neglecting the systemic forces that drive those behaviours (Monnat, 2017; Segal et al., 2017). Second, this moralising discourse strips DoD of their urgency. When deaths from these causes are read as a reflection of bad decisions or flawed morality, it becomes easier for institutions, and society more broadly, to dismiss them as inevitable or self-inflicted. In doing so, symbolic violence does not just misrepresent the causes of DoD, it actively undermines the conditions necessary for collective care, structural change, and political accountability. Taken together, the findings suggest that symbolic violence helps sustain geographic inequalities in DoD not only by obfuscating their political and economic determinants, but by shaping what is seen as morally and politically actionable. By positioning DoD as products of individual failure, symbolic violence forecloses structural explanations and limits the social imagination necessary for reform.

### 4.2 Territorial stigma

The findings of this study highlight how territorial stigma functions as a key mechanism of symbolic violence, compounding the structural and emotional harms experienced in post-industrial communities. As participants described, towns like Middlesbrough and South Tyneside are widely viewed as places of failure, dysfunction, and social deviance, perceptions reinforced through media portrayals, political rhetoric, and everyday discourse. What participants have described is territorial stigma (Wacquant, 2007), a phenomenon that is well documented in prior ethnographic research in low-income communities and in the Teesside region, where Middlesbrough is located, specifically (Bambra, 2016; Garthwaite and Bambra, 2018; Wacquant et al., 2014; Kallin and Slater, 2014). This spatialised form of stigma does more than mark certain places as undesirable; it shapes how residents see themselves and are seen by others, undermining their sense of worth, belonging, and political visibility (Wacquant et al., 2014; Bambra, 2016). The territorial stigma described by participants is a form of symbolic violence, naturalising these negative representations, encouraging both outsiders and residents to interpret structural disadvantage as a reflection of local culture, personal failings, and moral deficiency. In this way, symbolic violence in the form of territorial stigma not only legitimises the neglect of these communities but also displaces responsibility for suffering onto those who experience it.

This internalisation of spatial stigma has serious consequences for how social problems are understood and addressed. As participants indicated, growing up in a stigmatised area fostered shame and a sense of social exclusion, which shaped their expectations for education, employment, and overall life chances. These beliefs echo existing research showing that territorial stigma reduces political agency, diminishes access to services, and contributes to feelings of abandonment and despair (Kirkness and Tijé-Dra, 2017; Tyler and Slater, 2018). By obscuring structural causes and locating blame within communities themselves, territorial stigma operates as a cultural mechanism of obfuscation that sustains inequality while appearing apolitical. Territorial stigma contributes to a powerful cultural logic that justifies structural inequality: not only are these places seen as undeserving of investment, but the people within them are viewed as complicit in or responsible for their own marginalisation. As such, the findings of this study indicate that addressing inequalities in DoD in the context of stigmatised places requires more than economic renewal, it demands a fundamental shift in how places and populations are imagined, represented, and valued.

The findings of this study surrounding the role of territorial stigma in sustaining geographic inequalities in DoD align with and extend insights from the literature on moral geographies, which interrogates how spatial inequalities are legitimised through normative judgments about place (Smith, 2000; Valentine, 2014). Territorial stigma operates as a moralising discourse that casts post-industrial communities not only as economically marginal, but as sites of cultural and behavioural failure. As a result, structurally produced inequalities, such as those in rates of DoD, are rendered less visible or less urgent, reframed as the predictable outcomes of living in morally devalued spaces rather than as the products of systemic disinvestment (Anderson, 2013; Slater, 2018). This spatial moralisation contributes to what Garthwaite (2016) and Tyler (2013) describe as a politics of dehumanisation, whereby the suffering of stigmatised populations is normalised or dismissed. These findings demonstrate how symbolic violence operates through the internalisation of spatial stigma, reinforcing the idea that deprivation is rooted in place and character rather than structural neglect.

### 4.3 Obfuscation of root causes

The findings of this study support the view that charitable services such as foodbanks and community kitchens, while essential to survival for many, have become emblematic of systemic failure and state withdrawal. Previous ethnographic research conducted in Teesside has raised similar concerns (Garthwaite et al., 2015), and such criticisms have been levied by other academics as well (Riches, 2002; Schrecker and Bambra, 2015). Rather than being seen as evidence of a caring society, foodbanks were described by participants as evidence of political abandonment, a point echoed in Garthwaite’s (2016) analysis of the “normalisation” of emergency food provision in Teesside. What was once understood as a stopgap measure has become a routine feature of life in communities experiencing chronic deprivation. As participants noted, the very need for foodbanks in a wealthy nation signalled that the welfare state no longer guarantees basic material security.

This sense of abandonment was equally evident in participants’ experiences with the UK benefits system. Benefits were described as difficult to access, arbitrarily punitive, and emotionally degrading. Participants spoke of confusing bureaucratic processes, long wait times, and a culture of suspicion that left them feeling demoralised and stuck in a cycle of benefits management. These accounts resonate with existing research that has documented how post-2010 welfare reforms, particularly the rollout of Universal Credit and the expansion of benefit sanctions, have not only deepened poverty but intensified stigma and psychological distress (Wickham et al., 2020; Barr et al., 2016; Beatty et al., 2013; Wamsley, 2024). In this context, the benefits system functions not as a meaningful route out of poverty, but as another mechanism that symbolically and materially reinforces it. It provides just enough support to obscure the extent of deprivation and to offer hope to those in need of support, while simultaneously punishing those who rely on it through surveillance, conditionality, and moral judgement; in doing so, it reinforces the structural drivers of DoD. This dynamic has been described as a form of cruel optimism (Berlant, 2011). Individuals are encouraged to hope that navigating Universal Credit will eventually lead to stability, even as these mechanisms reinforce the precarity they are meant to alleviate (England, 2025). The affective promise of care masks the structural withdrawal of the state, luring people into attachments that are emotionally and materially unsustainable. England (2025) argues that the consequences of cruel optimism in poverty management is a population suspended in a holding pattern, encouraged to remain hopeful, but effectively immobilised, where coping becomes a substitute for transformation, and survival is mistaken for progress.

Together, the rise of charitable provision and the cruel optimism of welfare policy form part of a broader political strategy of displacement. Initiatives such as the “Big Society” reframed the erosion of state support as community empowerment, while austerity measures hollowed out local infrastructure and social protections (Cooper and Whyte, 2017; Smith and Jones, 2015), particularly in the North of England where a greater proportion of the population was reliant on such protections (Beatty and Fothergill, 2018). This study demonstrates how symbolic violence is central to this process: it moralises poverty, frames structurally induced suffering as individual failure, and legitimises minimal, conditional assistance. As participants made clear, reliance on food-banks and an antagonistic benefits system is not empowering, it is evidence of systemic abandonment, repackaged as care. These forms of support do not address the root causes of DoD, such as poverty and inequality; rather, they obscure them, offering the appearance of action while further entrenching deprivation. This symbolic performance diverts attention from the structural drivers of DoD by promoting narratives of personal responsibility and moral deficiency, which justify the status quo and foreclose demands for meaningful reform. In this light, both charity and the benefits system function not as solutions, but as tools through which social inequity is managed, concealed, and depoliticised. This process legitimises the conditions that produce DoD and allows such conditions to further proliferate. In this way, symbolic violence functions by masking the structural origins of despair, offering minimal forms of support that depoliticise suffering while reinforcing the very inequalities they appear to address.

### 4.4 Strengths and limitations

This study is grounded in the beliefs and of people living in areas most acutely affected by geographic inequalities in DoD, providing an important perspective that is often missing from research in this area. By relying on the voices of community members in Middlesbrough and South Tyneside, the study offers novel insight into how structural determinants and the inequalities they produce are justified and sustained through everyday public discourse and social narratives. A key contribution of this study is the use of symbolic violence as a conceptual framework, which reveals how internalised stigma, moralisation, and territorial shame function to depoliticise suffering and reinforce structural disadvantage. In doing so, the study makes a significant theoretical contribution by demonstrating how symbolic violence operates as a mechanism for sustaining geographic inequalities in rates of DoD in the English context.

While interpreting the findings of this study it is important to consider its limitations. Its cross-sectional design captured participant narratives at a single point in time, which constrains the ability to explore how symbolic violence and its effects on DoD inequalities may evolve or shift in response to changing policy, economic, and social conditions. A longitudinal approach would offer greater insight into how these cultural and structural dynamics are sustained or resisted over timeThe study’s focus is context specific, and symbolic violence may function differently in other settings. It remains unclear how symbolic violence interacts with DoD inequalities in other areas, particularly those with differing cultural, economic, or political contexts such as the US, where rates of DoD are considerably higher. Further research in varied geographic settings is therefore needed to examine whether similar mechanisms are at play, or whether symbolic violence takes different forms in sustaining these inequalities elsewhere.

## 5 Conclusion

The findings of this study help to explain why DoD are disproportionately concentrated in post-industrial communities. The evidence base surrounding the determinants of DoD points to structural forces such as poverty, economic exclusion, and policy failure (Case and Deaton, 2020; King et al., 2022; Beseran et al., 2022); however, the narratives presented here point to a complex interplay of structural neglect and cultural framing. As seen in this study, symbolic violence shifts the focus away from these root causes by moralising suffering and attributing it to individual behaviours, by stigmatising the places and people most affected, and by providing the appearance of taking action to address engrained inequalities. This reframing limits the political and public appetite for systemic interventions, contributing to the persistence of the broader social inequities that produce DoD. By framing addiction and mental illness, and their determinants as products of personal weakness, society avoids confronting the structural and policy-driven determinants of DoD. It is precisely this naturalisation of health inequality, the sense that these deaths are unfortunate but somehow deserved or inevitable, that symbolic violence helps to illuminate. It reveals the cultural and institutional processes that obscure structural harm and make deeply entrenched geographic inequalities in DoD appear normal, justified, and unchangeable.

In this context, the persistence of inequalities in DoD is not simply a matter of exposure to risk factors, but also of the cultural and institutional responses to suffering. The symbolic violence described by participants obscures the role of social determinants such as inequity, austerity, and place-based marginalisation in creating inequalities in DoD, while simultaneously ensuring that those affected are blamed for their own suffering. This dynamic helps explain why DoD inequalities remain so deeply entrenched: the systems ostensibly designed to protect and support vulnerable populations instead reproduce harm through stigma, neglect, and moral judgement. Addressing inequalities in DoD therefore requires more than better services, it demands challenging the cultural narratives that frame suffering as personal failure and health inequality as inevitable.

## Figures and Tables

**Table 1:** Participant demographic information.

Category	Middlesbrough (Number of Participants)	South Tyneside (Number of Participants)
Gender		
Male	9	9
Female	7	5
Age		
18−24	1	0
25−34	2	1
35−44	2	0
45−54	3	1
55−64	3	8
65+	5	4
Highest Level of Education Completed		
Primary school	5	2
Secondary school up to 16 years	4	8
Higher or secondary or further education (A-levels, BTEC, etc.)	2	3
College, university, post-graduate degree	2	1
Prefer not to say	3	0
Employment Status		
Unemployed	7	9
Part-Time	2	0
Full-Time	2	1
Retired	5	4
Years Lived in Town		
1−5 Years	1	1
6−10 Years	2	3
11−15 years	2	0
15+	11	10
Total Participants	16	14

## Data Availability

To ensure participant anonymity, the data underpinning this article are not publicly available.
